# Differences in airborne stability of SARS-CoV-2 variants of concern is impacted by alkalinity of surrogates of respiratory aerosol

**DOI:** 10.1098/rsif.2023.0062

**Published:** 2023-06-21

**Authors:** Allen Haddrell, Mara Otero-Fernandez, Henry Oswin, Tristan Cogan, James Bazire, Jianghan Tian, Robert Alexander, Jamie F. S. Mann, Darryl Hill, Adam Finn, Andrew D. Davidson, Jonathan P. Reid

**Affiliations:** ^1^ School of Chemistry, Cantock's Close, University of Bristol, Bristol, UK; ^2^ School of Cellular and Molecular Medicine, University of Bristol, Bristol, UK; ^3^ School of Population Health Sciences, University of Bristol, Bristol, UK; ^4^ Bristol Veterinary School, University of Bristol, Langford House, Langford, Bristol, UK

**Keywords:** variants, SARS-CoV-2, aerosol, pH, aerosol transmission, longevity

## Abstract

The mechanistic factors hypothesized to be key drivers for the loss of infectivity of viruses in the aerosol phase often remain speculative. Using a next-generation bioaerosol technology, we report measurements of the aero-stability of several SARS-CoV-2 variants of concern in aerosol droplets of well-defined size and composition at high (90%) and low (40%) relative humidity (RH) upwards of 40 min. When compared with the ancestral virus, the infectivity of the Delta variant displayed different decay profiles. At low RH, a loss of viral infectivity of approximately 55% was observed over the initial 5 s for both variants. Regardless of RH and variant, greater than 95% of the viral infectivity was lost after 40 min of being aerosolized. Aero-stability of the variants correlate with their sensitivities to alkaline pH. Removal of all acidic vapours dramatically increased the rate of infectivity decay, with 90% loss after 2 min, while the addition of nitric acid vapour improved aero-stability. Similar aero-stability in droplets of artificial saliva and growth medium was observed. A model to predict loss of viral infectivity is proposed: at high RH, the high pH of exhaled aerosol drives viral infectivity loss; at low RH, high salt content limits the loss of viral infectivity.

## 1. Introduction

**Figure 1.**
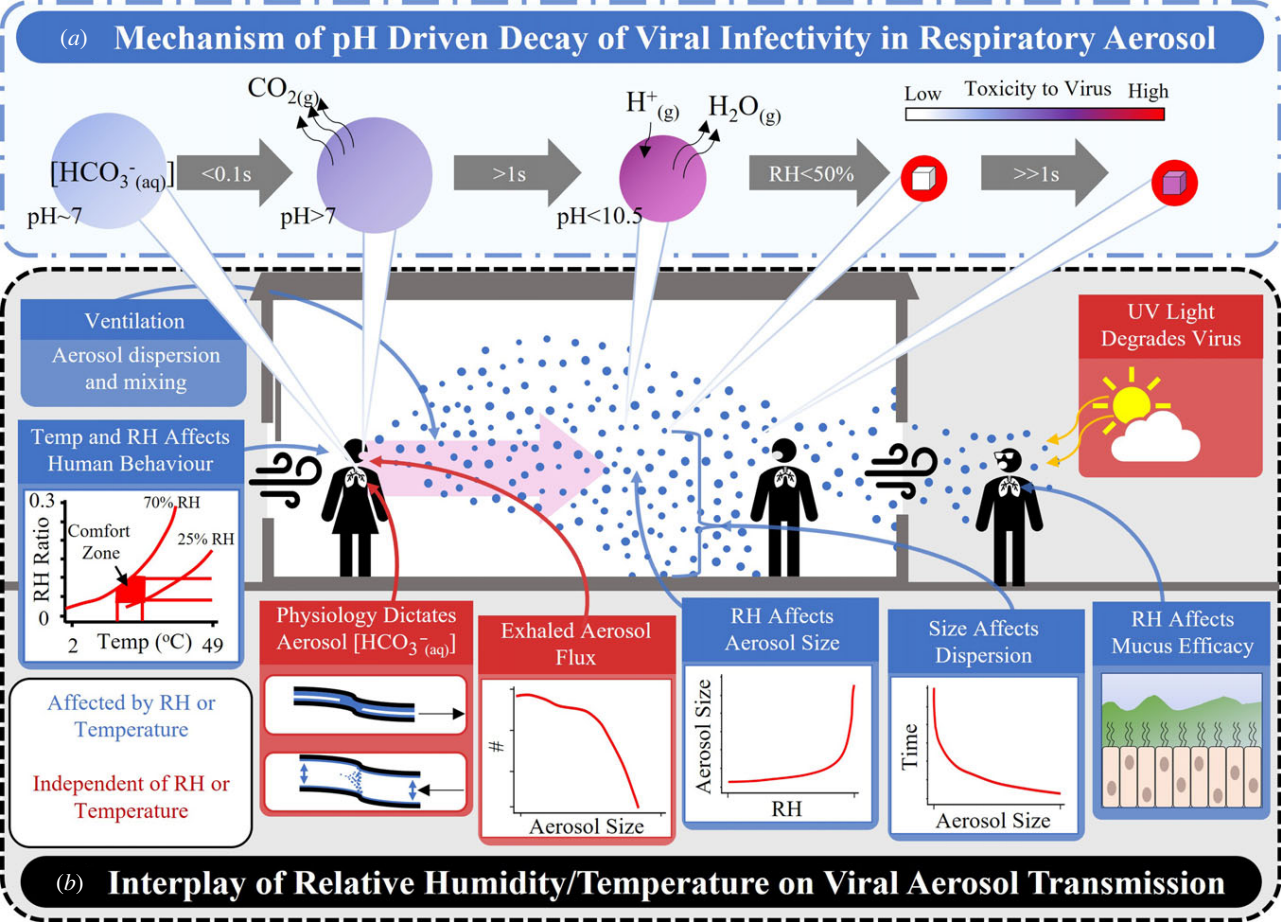
Brief summary of the interplay of factors involved in aerosolized viral transmission. (*a*) Hypothesized decay mechanism to explain the loss of viral infectivity in the aerosol phase [6]. At high relative humidity, the loss of viral infectivity is driven primarily by the high pH of the liquid aerosol droplet. At low humidity, the droplet may effloresce, resulting in two distinct microenvironments. Here, we propose that the organic fraction is harmful to the virus while the crystalline fraction is not. (*b*) Summary of the factors that influence the aerosol transmission of an aerosolized virus.

The dominant driver of the coronavirus disease 2019 (COVID-19) pandemic is the spread of severe acute respiratory syndrome coronavirus-2 (SARS-CoV-2) via airborne droplets and aerosols [1,2]. Identifying aerosols as the dominant vector by which COVID-19 is spread has helped to guide effective mitigation strategies, such as the use of masks [3], ventilation [4], air purifiers [4] and social distancing [5]. The complexities associated with the numerous factors that affect the transmission of an aerosolized virus are both well appreciated and often confounding (figure 1). These factors cross a broad range of disciplinary boundaries, including: biological (e.g. innate and adaptive immune defences [7], aerosol production [8,9], viral load per aerosol droplet, ability of mucous membranes to limit infection at low relative humidity [10]); human behaviour (e.g. air travel, indoor crowding, public transport, vaccination uptake and school attendance); government policy (e.g. mask mandates or quarantines [11]); and physical and environmental parameters that can affect viral longevity in the aerosol phase [6] (e.g. temperature, relative humidity (RH) [12], air movement [13] and UV light [14]). Although this list is by no means exhaustive, it demonstrates some of the many factors that can affect the transmission of an aerosolized virus. Moreover, it highlights the tremendous challenges associated with trying to reconcile all the different factors in models of viral transmission. These co-factors make the identification of the physico-chemical parameters that affect the airborne transmission of a virus impossible to elucidate from epidemiological data alone.

Changes in environmental conditions, such as RH and temperature, can have conflicting impacts on different stages in the transmission of an aerosolized virus (figure 1*b*). For example, at low RH the upper respiratory tract may become more vulnerable to infection through desiccation of mucous membranes and reduction of viral removal by mucociliary clearance (MCC), resulting in more infection and onward transmission [10,15]. Conversely, SARS-CoV-2 has been shown to lose greater than 50% of its infectivity near instantaneously (less than 5 s) in the aerosol phase at low RH, while remaining infectious much longer at higher RH [6,16]. Thus, low RH may both reduce and increase viral transmission by affecting both aerosol viral load and capacity to cause infection. Given this often conflicting impact of different factors, it is unlikely that any single one is entirely dominant in influencing transmission of the virus across a broad range of conditions. Rather, some will predominate over others under specific sets of conditions.

Animal models have been employed to study aerosolized viruses, including SARS-CoV-2 [17,18]. Guinea pig models have shown that transmission of influenza virus is affected both by temperature and humidity [12]; the highest transmission rates are at the lowest and highest RH, and transmission rates fall as temperature is increased [19]. More recently, a golden Syrian hamster model (GSH) has been adapted to study airborne transmission of SARS-CoV-2 [20,21]. Transmission was independent of RH and temperature following prolonged exposure over an ‘optimal time period’ (16 to 48 h). Over shorter time periods (less than 1 h) transmission rates were observed to be both RH and temperature dependent and increased at higher humidity and temperature [22].

Epidemiological studies have reached conflicting conclusions about the effects of RH and temperature on the transmission of SARS-CoV-2 in human populations [23–25]. Notably, RH has been found to correlate both positively [26,27] and negatively [28–31] with the spread of COVID-19.

To remain infectious in the aerosol phase, a virus must be stable within the unique microenvironment of an exhaled droplet. The increase in aerosol pH following exhalation has been reported through measurements of exhaled breath condensates [32–34] and results from the flux of CO_2_ from the aerosol droplet at the point of exhalation, originating in the solution phase as dissolved ${\mathrm{HCO}}_{3(\mathrm{aq})}^{-}$. This loss of ${\mathrm{HCO}}_{3(\mathrm{aq})}^{-}$ results in the pH of exhaled aerosol increasing from approximately neutral (approx. 7) when in the lung to highly alkaline (approx. 11) [6,35] following exhalation. When compared with all other environmental aerosols, this process is unique to respiratory aerosols as they are formed in a CO_2_ -rich environment [6]. Once aerosolized and following the increase of pH as a result of the flux of CO_2_ , the pH will slowly decrease due to the presence of condensable acidic species in the air [36]. The exact rate at which this occurs is unclear, what is clear is that it is dependent on the size of the aerosol particle and RH as well as the levels of acid content in the air.

The aim of this study is to examine the decay profile of SARS-CoV-2 as a function of environmental parameters such as temperature, RH and particle and gas phase composition. The relationships between the physico-chemical properties of surrogates of exhaled aerosol with viral longevity are then explored further, providing insights into the mechanistic factors that regulate the survival of viruses while airborne. The role of pH sensitivity as a primary driver of the loss of viral infectivity in the aerosol phase is demonstrated through comparison of the bulk and aerosol survival of SARS-CoV-2 variants of concern (VOCs).

## 2. Material and methods

Details of virus strains and methodologies for viral/cell culture, viral infectivity quantification, bulk stability measurements and levitation measurements can be found in the electronic supplementary material.

## 3. Results

### 3.1. Longevity of the SARS-CoV-2 wild type and Delta variant of concern as a function of relative humidity

**Figure 2.**
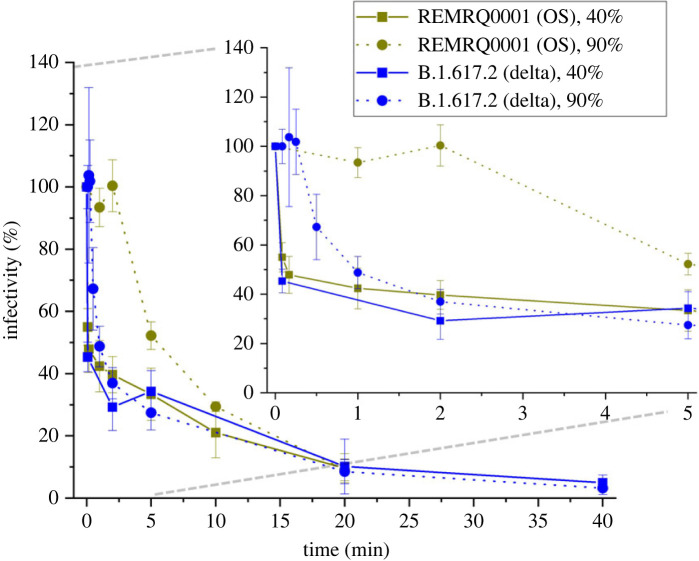
Infectivity of the SARS-CoV-2 OS virus and Delta VOC (OS and Delta) in the aerosol phase at moderate/low (40%) and high (90%) RH. The average number of levitations for each data point of the Delta VOC dataset is six, each levitation involving nine droplets with a mean number of viral particles per droplet of 4.4. Inset panel shows the same data for the first 5 min on an enlarged x-axis. When aerosol droplets containing the Delta VOC are levitated in a high (90%) RH airflow, a lag prior to the reduction in viral infectivity is observed, similar to that observed for the OS virus; the Delta VOC is found to show a much shorter lag period (approx. 15 s) than the OS virus (approx. 2 min).

The infectivity of the SARS-CoV-2 (ancestral Wuhan (original) virus (OS) and the Delta VOC) in aerosol droplets of minimum essential medium (MEM) + 2% fetal bovine serum (FBS) as a function of time (5 s–40 min) and RH (90% and 40%) are reported (figure 2). At moderate/low (40%) RH, the infectivity decay curves for the OS virus and the Delta VOC are the same within experimental uncertainty. Notably, there is an initial near instantaneous loss of over 50% of viral infectivity for both variants when efflorescence (spontaneous salt crystallization) occurs. Additionally, after longer times the survival at low RH shows a similar trend to high RH. This suggests that the efflorescence does not provide additional protection to the virus over time. The similarity in decay at 40% RH for both strains agrees with a recent study [16].

Collectively, the data presented in figure 2 demonstrate that the measurable differences in viral infectivity detected between the OS and Delta VOC in the aerosol phase across a broad range of RHs are seen primarily over a relatively short time scale (under 5 min as opposed to hours [37]). While this time scale may appear brief, it can be expected that an aerosol droplet could travel many metres in that time [38], and thus this difference may have an impact on viral transmission. For the Delta VOC, between 1 and 40 min, the infectivity at each time point is indistinguishable at the two RHs, by which time greater than 95% of the total virus infectivity is lost (infectivity at 40 min for 90% RH is 3.1 ± 2.0%, and for 40% RH is 4.9 ± 2.5%).

### 3.2. Aero-stability of SARS-CoV-2 variants correlates with sensitivity to alkaline pH

**Figure 3.**
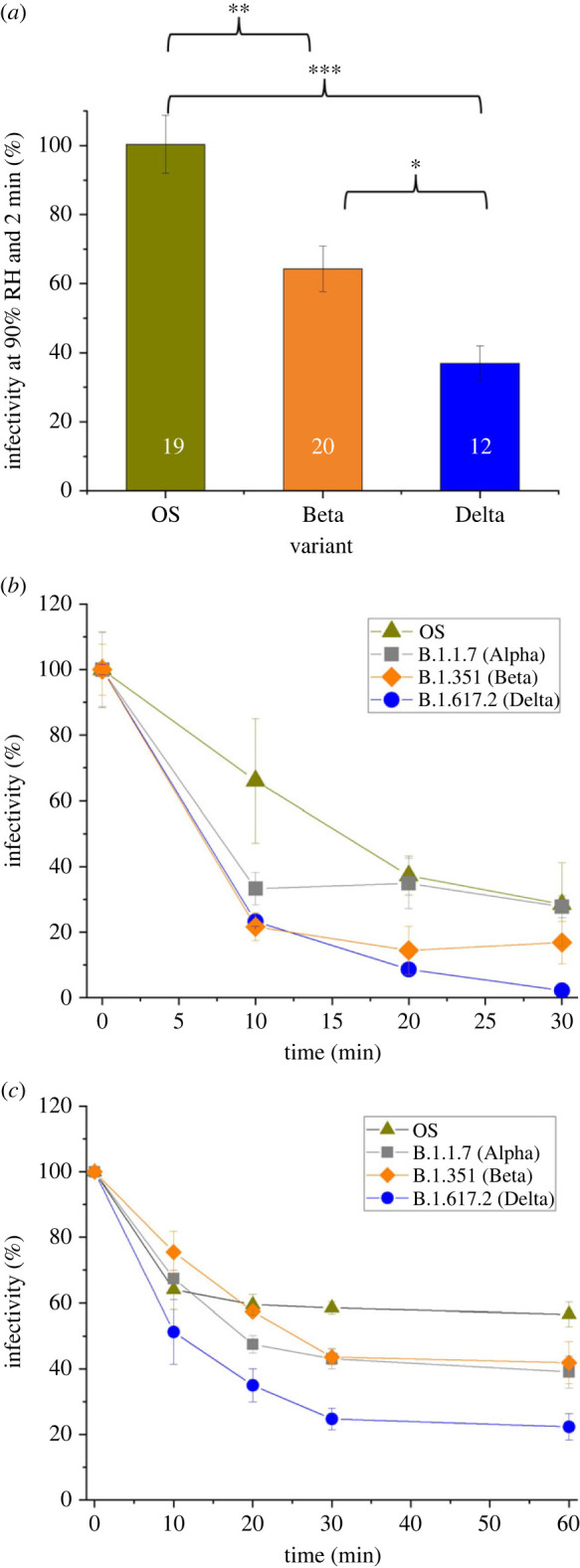
(*a*) SARS-CoV-2 VOCs exhibit differences in aero-stability over 2 min at high RH; the number in each bar indicates the number of individual levitations for each sample. Infectivity of multiple SARS-CoV-2 VOCs in MEM containing 2% FBS, pH = 11 after incubation in the bulk phase at room temperature as measured by (*b*) cytopathic effect and (*c*) immunostaining (pH = 7 was also measured for both, and no loss of infectivity was observed; data not shown). Statistical significance was assessed using a two-sample equal-variance *t*-test (*p ≤ 0.05, **p ≤ 0.005, and ***p ≤ 0.0005), error bars show standard error and lines are added to guide the eye.

The aero-stability of the Delta VOC was compared with the other SARS-CoV-2 VOCs reported previously [6] (electronic supplementary material, figure S2*a*, where infectivity of the OS virus, Alpha and Beta variants after 5 min of being in the aerosol phase for both moderate/low (40%) and high (90%) RH is compared with the Delta variant). When the RH is at 40% (associated with efflorescence), a similar survival fraction at 5 min is observed. However, when the RH is well above the deliquescence point of NaCl (RH 90%), the survival in the aerosol phase at 5 min follows a general reduction in aero-stability from VOCs Alpha through to Delta. Interestingly, the Delta variant is the first VOC to be identified as having a statistically significantly greater fall in stability while in the aerosol phase when compared with any other variant. When the time the virus is in the aerosol phase is shortened to 2 min, a general reduction in aero-stability from VOCs OS through to Delta is observed (figure 3).

To explore whether pH sensitivity of the VOCs drives the trend in figure 3*a* and electronic supplementary material, figure S2*a*, the loss of infectivity as a function of time at a pH of 11 (bulk solution) is reported in figure 3*b* and electronic supplementary material, figure S2*b*. These data are consistent with the hypothesis that the sensitivity to high pH correlates with the observed change in aero-stability. The correlation between decreased airborne virus stability and high pH in the bulk phase can be more clearly seen in electronic supplementary material, figure S2*b,* with the remaining infectivity after 20 min at pH 11 substantially lower for the Beta and Delta VOCs compared with the OS virus and Alpha VOC. The influence of high pH on infectivity was further confirmed using an immunoassay to detect SARS-CoV-2-infected cells directly rather than a cytopathic effect assay, which is potentially more subjective, and similar trends in viral stability are observed (figure 3*c* and electronic supplementary material, figure S2*c*). Biologically, pH can influence the transmission of SARS-CoV-2 in a myriad of ways [39], thus it is unsurprising to observe a pH-driven sensitivity.

### 3.3. Lowering acid content of the air dramatically reduces viral infectivity in the aerosol phase

**Figure 4.**
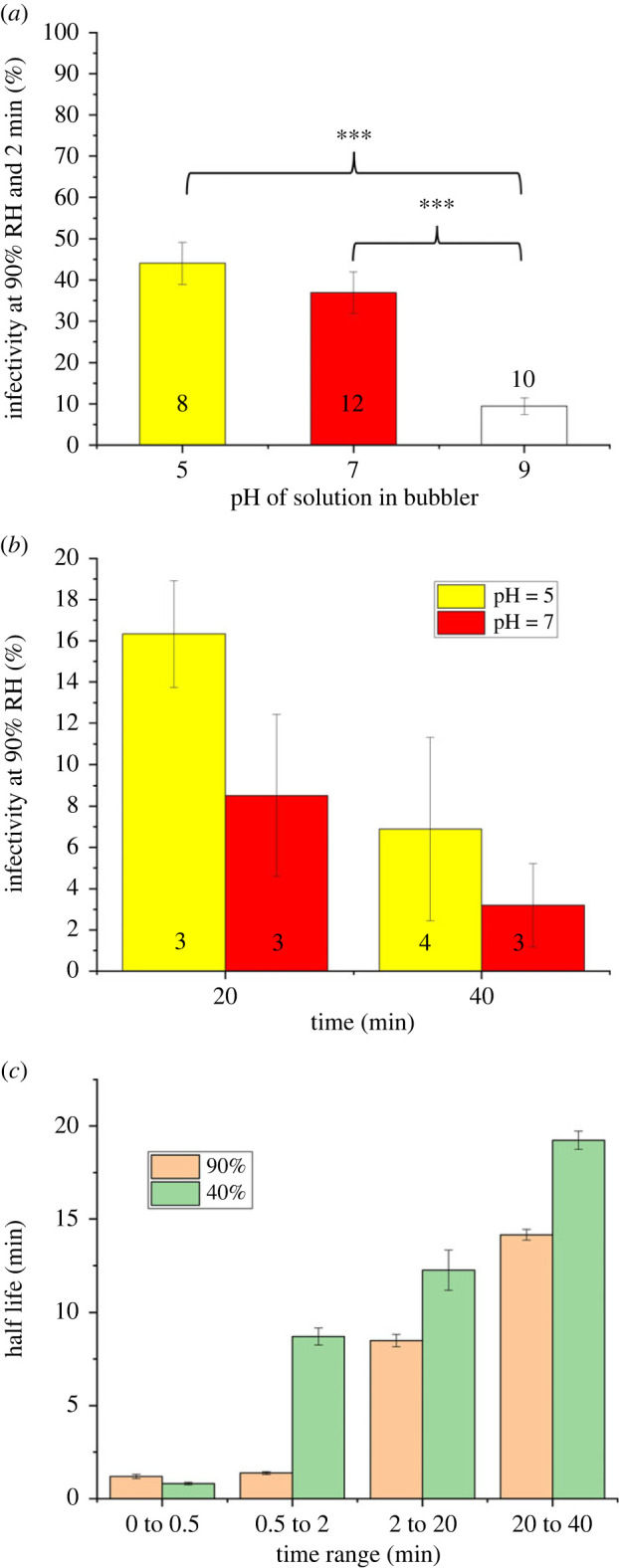
The effect that changing the acid content of the airflow over the levitated droplets has on virus infectivity of the Delta VOC as a function of pH over (*a*) short and (*b*) long periods of time. Numbers in each bar indicate the number of individual levitations for each set of conditions. (*c*) The half-life of the SARS-CoV-2 Delta VOC in the aerosol phase as a function of the time range over which the rate of loss was measured and RH. Statistical significance was assessed using a two-sample equal-variance *t*-test (***p ≤ 0.0005), and error bars indicate standard error.

In our previous study [6] we demonstrated that elevating the acid content of the air (specifically by adding CO_2_ ) increased the aero-stability of the virus with statistical significance. This was the result of the CO_2_ buffering the alkaline droplet in the same way that the CO_2_ in an incubator is used to buffer cell culture media. Broadly, to quantify the effect of the buffering capacity of the air surrounding the aerosol on viral longevity in this study, the overall acid content of the gas phase is manipulated through the addition and removal of nitric acid, and the effect on viral infectivity of the Delta VOC quantified (figure 4). Nitric acid is a commonly studied acid within the field of indoor air pollution [40].

Increasing the acid content of the air, specifically through adding HNO_3(aq)_ to the aqueous solution used to humidify the airflow, is found to have a minimal effect on virus stability (figure 4*a*). Based solely on vapour pressure, a pH 5 solution of nitric acid (1.9 × 10^−5^ M) would result in a gas phase with a HNO_3_ concentration of approximately 50 ppb [35]; however, given the propensity for HNO_3_ to condense onto surfaces, the precise concentration is not known. Previous studies have shown that SARS-CoV-2 is inactivated at an increasingly higher rate as the pH of the solution containing the virus is lowered below 7 [41]. The minimal impact of introducing additional acidity to the gas phase on the aerostability of the virus, indicates that the pH of the aerosol is likely to be above 7 immediately following generation. Therefore, the presence of condensable nitric acid can be hypothesized to be countering the strongly alkaline conditions formed immediately on droplet generation. This is expected since the rate of acid uptake, which is a function of RH and droplet size, is much too slow to limit the initial pH flux from CO_2_ loss; the result is that the pH buffering of an exhaled aerosol is asymmetric. This dynamic is intrinsically associated with all respiratory aerosols and is further amplified by the relatively large droplet size used in this study [42]. Accordingly the acid buffering capacity driving an increase in the aero-stability of the virus may be more consequential in environments where the droplet sizes are both smaller in diameter [9] and in air with elevated acid content (e.g. CO_2_ ), such as classrooms [43] and crowded offices [44], and laboratory studies of aero-stability using rotating drums [37].

When NaOH is used to adjust the pH of the bubbler to 9, a significant reduction of viral infectivity is observed (figure 4*a*) with approximately 90% loss in just 2 min. For context, it has been reported that high UV (simulated sunlight) intensity takes 8 min to produce a similar loss of infectivity of virus in the aerosol phase [14]. It is notable that NaOH is non-volatile, meaning that the pH = 9 solution in the bubbler results in no additional component being added to the air that could cause the measured drop in viral infectivity. Rather, the bubbler is removing any trace of condensable acidic species from the air, effectively reducing the buffering capacity of the air reaching the levitated droplets/particles. Thus, as compared with the rate of loss described in the literature (90% loss of infectivity in 6.8 min by sunlight) [14], the active removal of acidic indoor air components would be expected to remove the infectious virus from the aerosol phase in approximately a quarter of the time. The absence of an acidic buffering capacity of ambient air causes the virus to decay faster as the droplet pH remains higher for longer.

After 20 and 40 min of being in the aerosol phase, the amount of infectious virus within the droplets levitated in the acidified air is double that of the air passed through neutral pH water (figure 4*b*). This suggests that the effect of increased acid content in the air may be more impactful over time.

To further explore the effect that aerosol ageing has on viral aero-stability, the half-life (assumed first-order decay) of SARS-CoV-2 over various time periods post aerosolization at high and low RHs is calculated (figure 4*c*). As the aerosol ages, the half-life of SARS-CoV-2 continually increases. This suggests that the conditions in the droplet change over time to become less detrimental to the virus (e.g. pH trending towards neutralization). It is well understood that in ambient conditions aerosols tend to become more acidic over time. The pH dynamics of respiratory aerosol are unusual in that the pH rapidly increases from pH 7 within the lung to approximately 11 immediately following exhalation. While the direction of the pH flux of respiratory aerosol is clear (peaking at approx. 11 after aerosolization before progressing down), the rate and magnitude is unclear. It will be a function of many parameters, including acid vapour concentration, RH and aerosol size. When the acid content of the air is increased, the half-life of SARS-CoV-2 between 20 and 40 min (assuming first order kinetics) in the presence of HNO_3_ is increased to 21.6 min, while in the absence of HNO_3_ the half-life in this time range is only 14.1 min (figure 4*c*). The degree of this neutralizing effect is time and droplet size-dependent, and thus may potentially have a significant role in super spreader events [45], where prolonged aero-stability becomes more consequential.

### 3.4. O_2_ , salt and solute effects do not drive loss of SARS-CoV-2 infectivity in the aerosol phase

**Figure 5.**
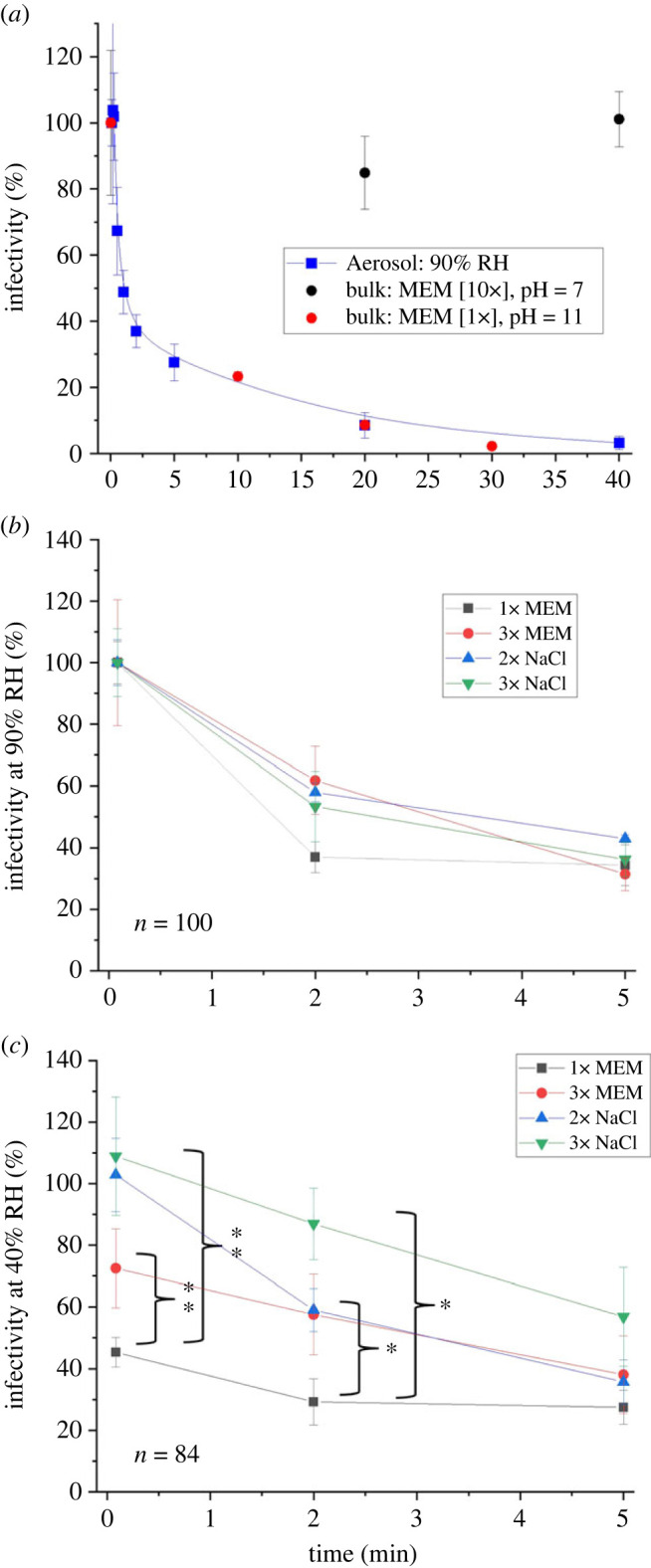
Systematically exploring characteristics of respiratory aerosols that may affect the loss of infectivity of the SARS-CoV-2 Delta VOC. (*a*) Comparison of the decay curves of SARS-CoV-2 in the aerosol phase with bulk measurements (high salt concentration and high pH). (*b*,*c*) Decay curves of SARS-CoV-2 in droplets whose initial composition has been spiked with additional solutes at (*b*) 90% and (*c*) 40% RH. ‘*n*’ indicates the number of individual levitations used to produce each figure. Statistical significance was assessed using a two-sample equal-variance *t*-test (*p ≤ 0.05 and **p ≤ 0.005), and error bars indicate standard error.

Multiple primary drivers of the loss of viral infectivity in the aerosol phase have been proposed, among which the most commonly reported are acidic pH [46], temperature, oxidative stress [47] and high solute (specifically salt) concentrations [48]. The extent to which these various factors drive the loss of viral infectivity were systematically tested (figure 5 and electronic supplementary material, figure S3).

Many studies have suggested that the high solute (e.g. salt) concentration reached in the aerosol phase reduces viral stability [46–49]. Below the deliquescence point (RH 75%), the salt concentration in the aerosol can reach supersaturation, a range not achievable in bulk solution. However, at a high relative humidity (90% RH), the salt concentration within an aerosol droplet can readily be replicated in bulk phase studies. This means that the hypothesis that the high salt concentration is driving the loss observed in figure 2 can be investigated readily in the bulk phase. No loss of viral infectivity is observed for the Delta VOC as a result of the solute concentration over the time periods (less than 60 min) relevant for virus aero-stability at high RH (figure 5*a*). Rather, the decay profile in the aerosol phase correlates very well with that of SARS-CoV-2 in the bulk phase at pH 11, indicating that aerosol pH is a critical factor in the observed loss.

There is evidence that adding oxidative components to the air, such as ozone, increases the loss of viral infectivity in the aerosol phase [50] but it is less clear whether oxidative damage from ambient air alone can drive such losses [47]. It has been reported [51] that switching the gas from air to nitrogen had no effect on the aero-stability of four different viral variants across a broad range of RH. A similar result is reported in this study (electronic supplementary material, figure S3*a*), where replacement of the flow of ambient air with nitrogen caused no significant change in viral aero-stability over the times studied. These data suggest that oxidative damage is not affecting viral infectivity over the time periods and conditions reported here.

The importance of ‘solute effects’ (i.e. the high concentration of all the solutes in the aerosol phase) on virus aero-stability is explored. The initial concentrations of all of the solutes (3× MEM) and just the NaCl fraction (2× and 3× NaCl) are adjusted and the effect on aero-stability is shown in figure 5*b*. At high RH, higher initial solute concentration, either total or just the NaCl component, has no significant effect on the decay in infectivity in the aerosol phase.

Collectively, the data shown in figure 5*a*,*b* and electronic supplementary material, figure S3, suggest that at humidities above efflorescence/deliquescence, neither oxidative stress, nor the concentration of salt nor of any of the other solutes are driving the loss of viral infectivity. Rather, it appears that the loss of viral infectivity above efflorescence is primarily driven by the high pH of aerosol following loss of the ${\mathrm{HCO}}_{3(\mathrm{aq})}^{-}$ buffer.

While the dissolved salt in the droplet was not found to influence viral stability at high RH, it was observed to have an indirect effect through efflorescence at moderate/low RH (rapid loss at 40% RH, figure 2). The efflorescence event produces two microenvironments within the droplet; we hypothesize that the virus in the soluble/organic fraction of the aerosol will rapidly lose its infectivity while the virus in the salt crystal remains somewhat stable (figure 1*a*). This hypothesis was tested by quantifying the effect on viral aero-stability of changing the relative abundance of inorganic and organic solutes.

#### 3.4.1. Initial total solute concentration has a moderate effect on aero-stability at low relative humidity

For droplets formed from a 1 : 1 mixture of the virus stock (Delta VOC) with 5× MEM + 10% FBS, there is a moderate, yet significant, increase in survival at moderate/low RH (figure 5*c*). This effect is readily explained by the paradigm described in figure 1*a*. Increasing the total starting solute concentration changes the absolute size of the NaCl crystal that can form through efflorescence. However, the proportion of the dried droplet that is crystalline is constant with the remaining organic fraction also constant. Thus, there is little consequence for stability.

#### 3.4.2. Increasing the NaCl solute fraction in the starting solute increases aero-stability at low relative humidity

From the paradigm described in figure 1*a*, we hypothesized that the survival of SARS-CoV-2 can be improved by increasing the proportion of the droplet that is crystalline salt, specifically the relative size of the NaCl crystal that is formed through altering the initial concentration of soluble NaCl. To test this hypothesis, the absolute amount and fraction of total solute that is NaCl is either doubled or tripled through spiking 12.5 and 25 µl of high salt concentration (3 g/10 ml) to 0.5 ml of the starting formulation.

At moderate/low RH, increasing only the amount of NaCl in the starting formulation causes a concentration-dependent increase in virus infectivity (figure 5*c*). Notably, the addition of the excess salt quenches the phenomenon of the near immediate loss of viral infectivity resulting from efflorescence. This demonstrates that the proportion of the droplet that is the organic fraction correlates with the loss of viral infectivity. Interestingly, the protective effect of the salt is found to be temporary and concentration dependent: after 5 min the protective effect of the salt crystal is no longer evident, and the decay profile is similar to that seen with high RH. Mucin has also been reported to produce a similar temporary protective effect [52].

The addition of excess salt results in no change in evaporation dynamics when the salt is doubled, and only a minor change when the concentration is tripled (electronic supplementary material, figure S3*b*). However, the addition of the salt results in a dramatic change in the crystalline structure produced (electronic supplementary material, figure S3*c*), where the higher salt produces a crystal structure more akin to that of pure NaCl (cubic) particle that is coated with an organic film.

Collectively, the data shown in figures 5 and electronic supplementary material, S3, support the paradigm described in figure 1*a*. The loss of viral infectivity is primarily driven by the alkaline pH of the aerosol, and not the high salt concentration (figure 5*a*) or oxidation (electronic supplementary material, figure S3*a*). At moderate/low RH (40%), by increasing only the salt component, the crystalline proportion of the NaCl crystal is dramatically increased, demonstrating that the loss of infectivity primarily occurs for virus contained within the organic fraction of the effloresced aerosol (figure 5*c*). The precise reason for this is unclear, as the physical characteristics of the organic fraction post efflorescence become difficult to define; for example, descriptions such as pH become challenging once the water content of the droplet becomes less than that of the organic fraction. While some work has begun to explore this in systems such as sea spray [53], further study into the chemical changes in a respiratory droplet specifically during efflorescence is needed.

The effect of temperature on the efflorescence-driven loss of viral infectivity was explored (electronic supplementary material, figures S4 and S5). Over short time periods (less than 30 s), temperature (5°C versus 20°C) was found to have a significant effect on aero-stability where the virus remains 100% infectious longer at lower temperatures (electronic supplementary material, figure S5).

### 3.5. Aero-stability of SARS-CoV-2 in artificial saliva is similar to that in growth media

**Figure 6.**
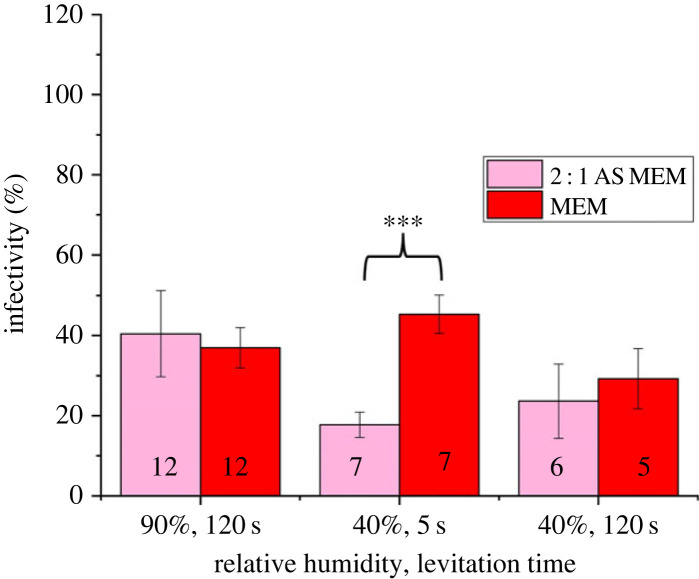
Aero-stability of the SARS-CoV-2 Delta VOC in droplets of artificial saliva (AS; 0.1% mucin) [54] and MEM across a range of relevant times and humidities. Numbers in each bar indicate the number of individual levitations for each sample. Statistical significance was assessed using a two-sample equal-variance *t*-test (***p ≤ 0.0005) while the error bars indicate standard error.

When considering only the parameters demonstrated in this study to dictate viral infectivity in the aerosol phase (pH and efflorescence), the chemical composition of artificial saliva and MEM are similar (electronic supplementary material, figures S6*a*,*b*) such that a similar decay rate would be expected. They both contain similar concentrations (by mass) of the chemical species understood to affect viral infectivity in the aerosol phase: salt (NaCl + KCl) and NaHCO_3_; note that the [${\mathrm{HCO}}_{3(\mathrm{aq})}^{-}$] in both solutions are very similar as they are both equilibrated with 5% CO_2_ . Consequently, it is expected that the decay dynamics of SARS-CoV-2 in the aerosol phase will be similar for both droplet types (figure 6).

Within the time periods and RH range where large variations in viral infectivity are observed (figure 2), changes in the droplet composition from MEM to artificial saliva results in a short-lived significant change in aero-stability at RHs below the efflorescence of NaCl (figure 6). This reduction in aero-stability at moderate/low RH (40%) is unsurprising given the relative lower quantity of salt in artificial saliva relative to MEM (electronic supplementary material, figure S6*a,b*). The reduced salt-to-organic ratio in the solute would produce a smaller salt crystal within the drying droplet, and thus would result in a lower aero-stability (figure 5*c*). Above efflorescence, no difference in aero-stability was observed for the Delta VOC in droplets of MEM and artificial saliva, agreeing with previous studies [14] and the data reported here (figure 5*b*). These data suggest that, given the underlying parameters that drive the loss of viral infectivity in the aerosol phase, growth medium can be considered a suitable proxy for exhaled aerosol if the salt concentration is considered, especially for short time periods (less than 2 min). Moreover, these data demonstrate the utility of the robust paradigm presented in figure 1*a* where the decay behaviour in other respiratory aerosols, such as deep lung fluid and nasal mucus, can be estimated.

## 4. Discussion

Between June 2021 and January 2022, the SARS-CoV-2 Delta variant (B.1.617.2) emerged to become the dominant variant of concern (VOC) of the COVID-19 pandemic [55]. There have been numerous biological theories proposed as to why this occurred. These range from increased transmissibility through, for example, mutations of the spike protein [56], a higher growth rate [57], immune pressure [58], or others. Whether the aero-stability of the virus itself played a role remains unknown. Given the rapid emergence of the Delta variant, it is surprising to find that the Delta variant is less aero-stable than the OS virus and other variants in the aerosol phase (RH > 40%). This highlights the importance of interpreting airborne stability data in the context of the complexities associated with the spread of an aerosolized virus (as described in figure 1). The data presented in figure 3 and electronic supplementary material, figure S2, suggest that the loss of aero-stability may be an evolutionary trade-off [59] for a more effective infection route following the inhalation/deposition event.

It has been hypothesized that as a respiratory droplet evaporates, the increase in salt concentration will drive the loss of infectivity [47]. At relative humidities below efflorescence, the proposed result is twofold: (i) the high concentration of salt would be removed from the solution phase, resulting in an organic fraction that is safe for the virus, and (ii) creating a crystal structure that can protect the virus. The result of this process is the ‘U-shaped’ association between RH and viral survival that is commonly reported [60]. If soluble salt concentration alone was driving the loss of viral infectivity, the relationships in figure 5*b*,*c* would not be observed. Succinctly, altering the ratio of the salt to organic fraction should not alter the decay dynamics if the loss of viral infectivity is driven by the high salt concentration. While it has been reported that the effloresced salt particle in a respiratory droplet will protect the virus [47,49], what has been overlooked entirely is the potential of the remaining organic fraction of the droplet to reduce the infectivity of the virus. The data shown in figures 2 and 5 suggest that the efflorescence event creates two distinct microenvironments in the droplet, one that is safe(r) for the virus, the other that is highly toxic and that has been made so through the combination of salt and water removal from the droplet (figure 1*a*). Moreover, the paradigm proposed here explains why any decay would occur below the efflorescence point.

It is well understood that SARS-CoV-2, being a respiratory virus, is largely spread indoors [4]. The reasons for this are plentiful, including closer proximity, low air change rates, and the absence of UV light, to name only a few. To this list, the study here shows that indoor air composition can influence the length of time that the virus remains infectious in the aerosol phase (figure 5*a*; removal of acid vapour rapidly lowers viral infectivity). The potential for acid vapour to buffer, and subsequently prolong viral aero-stability may affect disease transmission dynamics and mitigation strategies.

### 4.1. Potential implications on disease dynamics

The selection of cleaning agents aimed at reducing fomite transmission could influence how long SARS-CoV-2 remains infectious in the aerosol phase. For example, when bleach is exposed to open air it releases hypochlorous acid (HOCl, a volatile acid). HOCl has been measured in aerosol following the cleaning of a room [61]. Thus, while effective to control fomite spread of SARS-CoV-2, the use of bleach could inadvertently increase the length of time SARS-CoV-2 remains infectious in air through buffering the pH of the exhaled aerosol. The interplay between volatile acids produced from cleaning products with the survival SARS-CoV-2 in the aerosol phase should be explored further. The utility of the mechanistic paradigm (figure 1*a*) to predict the loss of viral infectivity is shown in this example, where seemingly irrelevant human behaviours, such as mopping with bleach, may have the potential to affect the airborne survival of a virus in a predictable way.

### 4.2. Implications on mitigation methods

The data reported in this study fully support three mitigation techniques: improved ventilation, social distancing and mask wearing; all are key to minimizing the number of infectious droplets reaching another person with the virus still infectious.

Aside from simply removing the amount of aerosol emitted and inhaled, when coupled with social distancing, mask usage will increase the time taken for the virus containing aerosol to reach another person. Given the 15 s ‘lag period’ (figure 2) in which no loss of infectivity was detected, the unencumbered aerosol could travel many metres, further demonstrating the utility of wearing a mask in reducing the general spread [62].

As it pertains to the pH of the aerosol, the overall power of ventilation to reduce the infectivity of SARS-CoV-2 in the aerosol phase is demonstrated in this study. By removing condensable acidic vapours from the air prior to introducing the airflow to the levitated droplets in the controlled electrodynamic levitation and extraction of bioaerosol onto a substrate (CELEBS) technique, we simulated the difference in survival of the virus in well-ventilated air and typical indoor air. Thus, by purifying the air in a room, specifically through lowering the total acid vapour content (e.g. CO_2_ , HNO_3_ ), the viral infectivity can be reduced four times faster by the clean air alone than by sunlight.

## Data Availability

Data from all of the figures can be found at: https://data.bris.ac.uk/data/dataset/1614tvzkl8×242styu6y64r7mb. The data are provided in electronic supplementary material [63].

## References

[1] Miller SL et al. 2021 Transmission of SARS-CoV-2 by inhalation of respiratory aerosol in the Skagit Valley Chorale superspreading event. Indoor Air. **31**, 314-323. (10.1111/ina.12751) 32979298 PMC7537089

[2] Tellier R. 2022 COVID-19: the case for aerosol transmission. Interface Focus. **12**, 20210072. (10.1098/rsfs.2021.0072) 35261731 PMC8831082

[3] Leech G et al. 2022 Mask wearing in community settings reduces SARS-CoV-2 transmission. Proc. Natl Acad. Sci. USA **119**, e2119266119. (10.1073/pnas.2119266119) 35639701 PMC9191667

[4] Morawska L et al. 2020 How can airborne transmission of COVID-19 indoors be minimised? Environ. Int. **142**, 105832. (10.1016/j.envint.2020.105832) 32521345 PMC7250761

[5] Jones B, Sharpe P, Iddon C, Hathway EA, Noakes CJ, Fitzgerald S. 2021 Modelling uncertainty in the relative risk of exposure to the SARS-CoV-2 virus by airborne aerosol transmission in well mixed indoor air. Build Environ. **191**, 107617. (10.1016/j.buildenv.2021.107617) 33495667 PMC7816614

[6] Oswin HP et al. 2022 The dynamics of SARS-CoV-2 infectivity with changes in aerosol microenvironment. Proc. Natl Acad. Sci. USA **119**, e2200109119. (10.1073/pnas.2200109119) 35763573 PMC9271203

[7] Cannell JJ, Vieth R, Umhau JC, Holick MF, Grant WB, Madronich S, Garland CF, Giovannucci E. 2006 Epidemic influenza and vitamin D. Epidemiol. Infect. **134**, 1129-1140. (10.1017/S0950268806007175) 16959053 PMC2870528

[8] Archer J et al. 2022 Comparing aerosol number and mass exhalation rates from children and adults during breathing, speaking and singing. Interface Focus **12**, 20210078. (10.1098/rsfs.2021.0078) 35261733 PMC8831083

[9] Gregson FKA et al. 2021 Comparing aerosol concentrations and particle size distributions generated by singing, speaking and breathing. Aerosol. Sci. Tech. **55**, 681-691. (10.1080/02786826.2021.1883544)

[10] Kudo E, Song E, Yockey LJ, Rakib T, Wong PW, Homer RJ, Iwasaki A. 2019 Low ambient humidity impairs barrier function and innate resistance against influenza infection. Proc. Natl Acad. Sci. USA **116**, 10 905-10 910. (10.1073/pnas.1902840116) PMC6561219 31085641

[11] Patel A, Patel S, Fulzele P, Mohod S, Chhabra KG. 2020 Quarantine an effective mode for control of the spread of COVID19? A review. J. Family Med. Prim. Care **9**, 3867-3871. (10.4103/jfmpc.jfmpc_785_20) 33110781 PMC7586567

[12] Lowen AC, Mubareka S, Steel J, Palese P. 2007 Influenza virus transmission is dependent on relative humidity and temperature. PLoS Pathog. **3**, 1470-1476. (10.1371/journal.ppat.0030151) 17953482 PMC2034399

[13] Mariam , Magar A, Joshi M, Rajagopal PS, Khan A, Rao M, Sapra BK. 2021 CFD simulation of the airborne transmission of COVID-19 vectors emitted during respiratory mechanisms: revisiting the concept of safe distance. ACS Omega **6**, 16 876-16 889. (10.1021/acsomega.1c01489) PMC8247784 34250347

[14] Schuit M et al. 2020 Airborne SARS-CoV-2 is rapidly inactivated by simulated sunlight. J. Infect. Dis. **222**, 564-571. (10.1093/infdis/jiaa334) 32525979 PMC7313838

[15] Courtney JM, Bax A. 2021 Hydrating the respiratory tract: an alternative explanation why masks lower severity of COVID-19. Biophys. J. **120**, 994-1000. (10.1016/j.bpj.2021.02.002) 33582134 PMC7879047

[16] Dabisch PA, Wood SP, Holland BP, Boydston JA, Beck KB, Green B, Biryukov J. 2022 Comparison of the survival of different isolates of SARS-CoV-2 in evaporating aerosols. Aerosol. Sci. Tech. **56**, 1-12. (10.1080/02786826.2022.2128712) PMC10698713 38075547

[17] Shou SY et al. 2021 Animal models for COVID-19: hamsters, mouse, ferret, mink, tree shrew, and non-human primates. Front. Microbiol. **12**, 626553. (10.3389/fmicb.2021.626553) 34531831 PMC8438334

[18] Kutter JS, de Meulder D, Bestebroer TM, Lexmond P, Mulders A, Richard M, Fouchier RAM, Herfst S. 2021 SARS-CoV and SARS-CoV-2 are transmitted through the air between ferrets over more than one meter distance. Nat. Commun. **12**, 1653. (10.1038/s41467-021-21918-6) 33712573 PMC7955093

[19] Lowen AC, Steel J. 2014 Roles of humidity and temperature in shaping influenza seasonality. J. Virol. **88**, 7692-7695. (10.1128/Jvi.03544-13) 24789791 PMC4097773

[20] Imai M et al. 2020 Syrian hamsters as a small animal model for SARS-CoV-2 infection and countermeasure development. Proc. Natl Acad. Sci. USA **117**, 16 587-16 595. (10.1073/pnas.2009799117) PMC7368255 32571934

[21] Sia SF et al. 2020 Pathogenesis and transmission of SARS-CoV-2 in golden hamsters. Nature **583**, 834. (10.1038/s41586-020-2342-5) 32408338 PMC7394720

[22] Ganti K, Ferreri LM, Lee CY, Bair CR, Delima GK, Holmes KE, Suthar MS, Lowen AC. 2022 Timing of exposure is critical in a highly sensitive model of SARS-CoV-2 transmission. PLoS Pathog. **18**, e1010181. (10.1371/journal.ppat.1010181) 35333914 PMC8986102

[23] Karmokar J, Islam MA, Uddin M, Hassan MR, Yousuf MSI. 2022 An assessment of meteorological parameters effects on COVID-19 pandemic in Bangladesh using machine learning models. Environ. Sci. Pollution R. **29**, 67 103-67 114. (10.1007/s11356-022-20196-z) PMC9073515 35522407

[24] Raines KS, Doniach S, Bhanot G. 2021 The transmission of SARS-CoV-2 is likely comodulated by temperature and by relative humidity. PLoS ONE **16**, e0255212. (10.1371/journal.pone.0255212) 34324570 PMC8321224

[25] Mecenas P, Bastos RTDM, Vallinoto ACR, Normando D. 2020 Effects of temperature and humidity on the spread of COVID-19: a systematic review. PLoS ONE **15**, e0238339. (10.1371/journal.pone.0238339) 32946453 PMC7500589

[26] Ganegoda NC, Wijaya KP, Amadi M, Erandi KKWH, Aldila D. 2021 Interrelationship between daily COVID-19 cases and average temperature as well as relative humidity in Germany (vol 11, 11302, 2021). Sci. Rep.-UK **11**, 18248. (10.1038/s41598-021-97830-2) PMC8163835 34050241

[27] He ZL et al. 2021 The influence of average temperature and relative humidity on new cases of COVID-19: time-series analysis. JMIR Public Health Sur. **7**, 231-244. (10.2196/20495) PMC7836910 33232262

[28] Ahlawat A, Wiedensohler A, Mishra SK. 2020 An overview on the role of relative humidity in airborne transmission of SARS-CoV-2 in indoor environments. Aerosol. Air Qual. Res. **20**, 1856-1861. (10.4209/aaqr.2020.06.0302)

[29] Ward MP, Liu YH, Xiao S, Zhang ZJ. 2021 Challenges in the control of COVID-19 outbreaks caused by the δ variant during periods of low humidity: an observational study in Sydney, Australia. Infect. Dis. Poverty **10**, 139. (10.1186/s40249-021-00926-0) 34937575 PMC8694908

[30] Robey AJ, Fierce L. 2022 Sensitivity of airborne transmission of enveloped viruses to seasonal variation in indoor relative humidity. Int Commun Heat Mass. **130**, 105747. (10.1016/j.icheatmasstransfer.2021.105747)

[31] Wang JY, Tang K, Feng K, Lin X, Lv WF, Chen K, Wang F. 2021 Impact of temperature and relative humidity on the transmission of COVID-19: a modelling study in China and the United States. BMJ Open **11**, e043863. (10.1136/bmjopen-2020-043863) PMC7893211 33597143

[32] Vaughan J et al. 2003 Exhaled breath condensate pH is a robust and reproducible assay of airway acidity. Eur. Respir. J. **22**, 889-894. (10.1183/09031936.03.00038803) 14680074

[33] Kostikas K, Papatheodorou G, Ganas K, Psathakis K, Panagou P, Loukides S. 2002 pH in expired breath condensate of patients with inflammatory airway diseases. Am. J. Resp. Crit. Care **165**, 1364-1370. (10.1164/rccm.200111-068OC) 12016097

[34] Hunt JF, Fang KZ, Malik R, Snyder A, Malhotra N, Platts-Mills TAE, Gaston B. 2000 Endogenous airway acidification – implications for asthma pathophysiology. Am. J. Resp. Crit. Care **161**, 694-699. (10.1164/ajrccm.161.3.9911005) 10712309

[35] Klein LK et al. 2022 Expiratory aerosol pH is determined by indoor room trace gases and particle size. Proc. Natl Acad. Sci. USA **119**, e2212140119. (10.1073/pnas.2212140119) 36037391 PMC9522367

[36] Tilgner A et al. 2021 Acidity and the multiphase chemistry of atmospheric aqueous particles and clouds. Atmos. Chem. Phys. **21**, 13 483-13 536. (10.5194/acp-21-13483-2021) PMC8525431 34675968

[37] van Doremalen N et al. 2020 Aerosol and surface stability of SARS-CoV-2 as compared with SARS-CoV-1. N Engl. J. Med. **382**, 1564-1567. (10.1056/NEJMc2004973) 32182409 PMC7121658

[38] Walker JS, Archer J, Gregson FKA, Michel SES, Bzdek BR, Reid JP. 2021 Correction to 'Accurate representations of the microphysical processes occurring during the transport of exhaled aerosols and droplets' (vol 7, p. 200, 2021). ACS Central Sci. **7**, 507-507. (10.1021/acscentsci.1c00220) PMC8006164 33791432

[39] Lobo VR, Warwicker J. 2021 Predicted pH-dependent stability of SARS-CoV-2 spike protein trimer from interfacial acidic groups. Comput. Struct. Biotechnol. J. **19**, 5140-5148. (10.1016/j.csbj.2021.08.049) 34490059 PMC8410215

[40] Weschler CJ, Brauer M, Koutrakis P. 1992 Indoor ozone and nitrogen-dioxide – a potential pathway to the generation of nitrate radicals, dinitrogen pentaoxide, and nitric-acid indoors. Environ. Sci. Technol. **26**, 179-184. (10.1021/es00025a022)

[41] Luo B et al. 2022 Acidity of expiratory aerosols controls the infectivity of airborne influenza virus and SARS-CoV-2. Environ. Sci. Technol. **57**, 486-497. (10.1101/2022.03.14.22272134)36537693

[42] Li M, Su H, Li G, Ma N, Poschl U, Cheng YF. 2019 Relative importance of gas uptake on aerosol and ground surfaces characterized by equivalent uptake coefficients. Atmos. Chem. Phys. **19**, 10 981-11 011. (10.5194/acp-19-10981-2019)

[43] Zemitis J, Bogdanovics R, Bogdanovica S. 2021 The study of CO_2_ concentration in a classroom during the Covid-19 safety measures. E3s Web Conf. **246**, 01004. (10.1051/e3sconf/202124601004)

[44] Rudnick SN, Milton DK. 2003 Risk of indoor airborne infection transmission estimated from carbon dioxide concentration. Indoor Air **13**, 237-245. (10.1034/j.1600-0668.2003.00189.x) 12950586

[45] Majra D, Benson J, Pitts J, Stebbing J. 2021 SARS-CoV-2 (COVID-19) superspreader events. J. Infect. **82**, 36-40. (10.1016/j.jinf.2020.11.021) 33245943 PMC7685932

[46] Yang W, Marr LC. 2012 Mechanisms by which ambient humidity may affect viruses in aerosols. Appl. Environ. Microb. **78**, 6781-6788. (10.1128/Aem.01658-12) PMC3457514 22820337

[47] Huynh E, Olinger A, Woolley D, Kohli RK, Choczynski JM, Davies JF, Lin KS, Marr LC, Davis RD. 2022 Evidence for a semisolid phase state of aerosols and droplets relevant to the airborne and surface survival of pathogens. Proc. Natl Acad. Sci. USA **119**, e2109750119. (10.1073/pnas.2109750119) 35064080 PMC8794803

[48] Yang W, Elankumaran S, Marr LC. 2012 Relationship between humidity and influenza A viability in droplets and implications for influenza's seasonality. PLoS ONE **7**, e46789. (10.1371/journal.pone.0046789) 23056454 PMC3463543

[49] Niazi S, Groth R, Cravigan L, He CR, Tang JW, Spann K, Johnson GR. 2021 Susceptibility of an airborne common cold virus to relative humidity. Environ. Sci. Technol. **55**, 499-508. (10.1021/acs.est.0c06197) 33332096

[50] Bayarri B, Cruz-Alcalde A, Lopez-Vinent N, Mico MM, Sans C. 2021 Can ozone inactivate SARS-CoV-2? A review of mechanisms and performance on viruses. J. Hazard. Mater. **415**, 125658. (10.1016/j.jhazmat.2021.125658) 33752085 PMC7955572

[51] Benbough JE. 1971 Some factors affecting survival of airborne viruses. J. Gen. Virol. **10**, 209. (10.1099/0022-1317-10-3-209) 4324730

[52] Alexander RW et al. 2022 Mucin transiently sustains coronavirus infectivity through heterogenous changes in phase morphology of evaporating aerosol. Viruses **14**, 1856. (10.3390/v14091856) 36146663 PMC9503081

[53] Dommer A et al. 2023 *Revealing the Impacts of Chemical Complexity on Submicron Sea Spray Aerosol Morphology*. Earth Space, and Environmental Chemistry. See https://chemrxiv.org/engage/api-gateway/chemrxiv/assets/orp/resource/item/63e702279da0bc6b33b6e6ac/original/revealing-the-impacts-of-chemical-complexity-on-submicron-sea-spray-aerosol-morphology.pdf

[54] Woo MH, Hsu YM, Wu CY, Heimbuch B, Wander J. 2010 Method for contamination of filtering facepiece respirators by deposition of MS2 viral aerosols. J. Aerosol Sci. **41**, 944-952. (10.1016/j.jaerosci.2010.07.003) 32226122 PMC7094656

[55] Parums DV. 2021 Editorial: Revised World Health Organization (WHO) terminology for variants of concern and variants of interest of SARS-CoV-2. Med. Sci. Monitor. **27**, e933622. (10.12659/MSM.933622) PMC8230247 34149046

[56] Liu Y et al. 2022 Delta spike P681R mutation enhances SARS-CoV-2 fitness over Alpha variant. Cell Rep. **39**, 110829. (10.1016/j.celrep.2022.110829) 35550680 PMC9050581

[57] Zhang J et al. 2021 Membrane fusion and immune evasion by the spike protein of SARS-CoV-2 Delta variant. Science **374**, 1353. (10.1126/science.abl9463) 34698504 PMC10763652

[58] McKeigue PM, McAllister DA, Hutchinson SJ, Robertson C, Stockton D, Colhoun HM. 2022 Vaccine efficacy against severe COVID-19 in relation to δ variant (B.1.617.2) and time since second dose in patients in Scotland (REACT-SCOT): a case-control study. Lancet Respir. Med. **10**, 566-572. (10.1016/S2213-2600(22)00045-5) 35227416 PMC8880999

[59] Goldhill DH, Turner PE. 2014 The evolution of life history trade-offs in viruses. Curr. Opin. Virol. **8**, 79-84. (10.1016/j.coviro.2014.07.005) 25087040

[60] Lin KS, Marr LC. 2020 Humidity-dependent decay of viruses, but not bacteria, in aerosols and droplets follows disinfection kinetics. Environ. Sci. Technol. **54**, 1024-1032. (10.1021/acs.est.9b04959) 31886650

[61] Wong JPS, Carslaw N, Zhao R, Zhou S, Abbatt JPD. 2017 Observations and impacts of bleach washing on indoor chlorine chemistry. Indoor Air **27**, 1082-1090. (10.1111/ina.12402) 28646605

[62] Schmitt J, Wang J. 2021 Quantitative modeling of the impact of facemasks and associated leakage on the airborne transmission of SARS-CoV-2. Sci. Rep.-UK **11**, 19403. (10.1038/s41598-021-98895-9) PMC8484595 34593891

[63] Haddrell A et al. 2023 Differences in airborne stability of SARS-CoV-2 variants of concern is impacted by alkalinity of surrogates of respiratory aerosol. Figshare. (10.6084/m9.figshare.c.6688649) PMC10282576 37340783

